# *Streptococcus suis*-induced mitophagy via Eno-HSP90AA1 interaction disrupts the blood–brain barrier

**DOI:** 10.1186/s13567-026-01860-5

**Published:** 2026-11-07

**Authors:** Xingxing Xiao, Yafang Zhou, Yike Fang, Stijn van der Veen, Xu-ai Lin, Yongliang Lou, Shuai Gao

**Affiliations:** 1 Wenzhou Key Laboratory of Sanitary Microbiology, Key Laboratory of Laboratory Medicine, Ministry of Education, School of Laboratory Medicine and Life Sciences, Wenzhou Medical University, Wenzhou, 325035 China; 2 Department of Microbiology, School of Medicine, Zhejiang University, Hangzhou, 310058 China

**Keywords:** Mitophagy, *Streptococcus suis* serotype 2, Enolase, HSP90AA1, blood–brain barrier

## Abstract

*Streptococcus suis* serotype 2 (SS2), a zoonotic pathogen associated with meningitis in humans and pigs, disrupts the blood–brain barrier (BBB) during infection, but underlying mechanisms remain inadequately elucidated. This study demonstrated that SS2 infection induced mitophagy in human brain microvascular endothelial cells (hBMECs), with Eno (Enolase) identified as the key bacterial protein responsible for the activation of PINK1-Parkin pathway. Using Co-immunoprecipitation, mass spectrometry, and confocal microscopy, we identified that Eno interacted with the host molecular chaperone HSP90AA1 via its N-terminal domain, necessitating the integrity of the second α-helix (residues 87–98). The interaction between Eno and HSP90AA1 may promote mitochondrial proteotoxic stress and misfolded protein accumulation, thereby contributing to mitophagy induction, while the loss of mitochondrial membrane potential (MMP) alone is insufficient to initiate this process. Importantly, Eno-induced mitophagy did not result in cell death but instead led to the degradation of the tight junction protein claudin-5 (CLDN5), thus compromising BBB integrity. Both in vitro BBB models and in vivo mouse infection studies substantiated the involvement of Eno-induced mitophagy in BBB disruption. These findings reveal a novel mechanism whereby SS2 Eno exploits host HSP90AA1 to activate mitophagy, triggering the degradation of CLDN5 to breach the BBB. This study provides new insights into SS2 pathogenesis and identifies the Eno-HSP90AA1-mitophagy axis as a potential therapeutic target for preventing bacterial meningitis.

**Supplementary Information:**

The online version contains supplementary material available at https://doi.org/10.1186/s13567-026-01860-5.

## Introduction

*Streptococcus suis* serotype 2 (SS2) is a significant zoonotic pathogen responsible for meningitis, arthritis, and sepsis in both humans and animals, posing a substantial threat to global public health and the swine industry [1, 2]. Human infection with SS2 primarily occurs through direct contact with infected pigs, exposure to contaminated pork products, or consumption of undercooked pork [3]. The global distribution of SS2 cases underscores its prevalence in Asia. Notably, China experienced two major outbreaks of SS2 in Jiangsu Province and Sichuan Province, in 1998 and 2005, respectively, resulting in numerous fatalities [4]. In recent years, SS2 human infection has also been reported in China; furthermore, it has emerged as the leading cause of bacterial meningitis in Southeast Asia, especially in Vietnam and Thailand [5–7].

Meningitis induced by SS2 represents a severe central nervous system infection, with its impact extending beyond the acute phase to include long-term sequelae such as hearing loss and cognitive dysfunction [3]. A critical step in the pathogenesis of SS2-induced meningitis involves the disruption of the blood–brain barrier (BBB) [8]. Once the integrity of the BBB is compromised, bacteria and a substantial influx of inflammatory mediators infiltrate the cerebrospinal fluid, facilitating bacterial dissemination within the central nervous system and eliciting a pronounced inflammatory response [9]. This intricate process is characterized by the interaction between diverse bacterial virulence factors and the host. The H-binding protein of SS2 influences cytoskeletal reorganization through its interaction with globotriaosylceramide, thereby facilitating bacterial traversal across the BBB [10]. The surface protein Sssp1 of SS2 augments bacterial adhesion and penetration capabilities at the BBB by binding to host cell vimentin [11]. Studies have demonstrated a correlation between Enolase (Eno) of SS2 and the increased permeability of the BBB during the infection process [12, 13]. Eno is integral to the glycolytic pathways, where it facilitates the conversion of 2-phosphoglycerate to phosphoenolpyruvate, which is subsequently converted to pyruvate to drive ATP synthesis [14, 15]. The functions and structures of enolases exhibit considerable diversity across different organisms. The Eno of SS2 is recognized as a multifunctional protein that not only contributes to glycolysis but also facilitates interactions between the bacterium and the host [14]. Previous research indicates that the interaction between Eno and host surface ribosomal protein SA (RPSA) induces apoptosis, ultimately leading to the breakdown of the BBB [12, 13].

Mitophagy, a selective form of autophagy, plays a crucial role in maintaining mitochondrial quality by removing damaged or dysfunctional mitochondria [16]. Various bacterial pathogens have evolved mechanisms to exploit mitophagy for their own benefits. *Listeria monocytogenes* hijacks host mitophagy through a novel receptor, NLRX1, to evade killing. This receptor directly interacts with LC3, a key autophagy protein, facilitating the induction of mitophagy [17]. Gonococcal PorB can induce mitophagy by activating the PINK1-NDP52/OPTN pathway [18]. Additionally, RNF213-mediated K63-linked polyubiquitination of PorB directly triggers its interaction with the autophagy receptor p62, thereby initiating mitophagy [18]. Upon cellular entry, *Brucella* induces an iron starvation response that enhances the stability and activation of HIF-1α. This process subsequently upregulates the expression of the mitophagy receptor BNIP3, ultimately resulting in the induction of mitophagy, which improves the ability of bacteria to transmit between cells and invade tissues [19]. Currently, however, it is still unclear whether SS2 can induce mitophagy.

The functional diversity of mitophagy plays a crucial role in modulating the interactions between pathogenic bacteria and the host, making it an effective target for the prevention and treatment of bacterial infection. In light of this, we aimed to ascertain whether SS2 is capable of activating mitophagy and to define the precise contribution of this process to SS2 infection. Here, we demonstrate that Eno of SS2 acts as an inducer of mitophagy for hBMECs via the PINK1-Parkin pathway. Although Eno can dissipate mitochondrial membrane potential (MMP), Eno-induced mitophagy necessitates the interaction between Eno and HSP90AA1, which may lead to the accumulation of misfolded proteins within mitochondria. Notably, Eno-induced mitophagy contributes to the degradation of claudin-5 (CLDN5), ultimately compromising the integrity of the BBB.

## Materials and methods

### Cell culture and transfection

hBMECs sourced from ScienCell Research Laboratories were cultured in Dulbecco’s Modified Eagle Medium (DMEM) with 10% fetal bovine serum (Gibco, C0232) at 37 °C in the presence of 5% CO_2_. To inhibit autophagy or proteasome, cells were pretreated with 3-MA (Selleck, S2767, 10 mM, 2 h), BafA1 (Selleck, S1413, 50 nM, 4 h), Mdivi-1 (Selleck, S7162, 20 μM, 2 h) and MG132 (Selleck, S2619, 5 μM, 6 h) respectively. To evaluate the functional properties of Eno, hBMECs at a density of 1 × 10^6^ were incubated with predetermined sublethal concentration (2 µg/mL) of purified recombinant Eno (rEno) and its variants for a duration of 4 h. For the analysis of time-dependent responses, cells were subjected to rEno at various time points: 0, 2, 4, and 6 h. Control groups consisted of cells treated solely with the PBS (Solarbio, P1020).

Transfections of hBMECs were conducted utilizing Lipo8000™ Transfection Reagent (Beyotime, C0533) in OptiMEM (Gibco, 31,985,062), incorporating 2 μg of plasmid DNA in 1 mL reaction mixture. After incubating with cells for 6 h, the mixture was replaced with fresh culture medium containing 10% fetal bovine serum.

### Bacterial strains

*Streptococcus suis* serotype 2 strain 05ZYH33 is a well-characterized highly virulent reference strain originally isolated from a human patient during the 2005 outbreak of infection in China. The strain was grown in Todd-Hewitt broth (Hopebio, HB0311-3) with an addition of 2% yeast extract (Oxoid, LP0021B) at 37 °C.

To generate the Δ*sly*, *eno::kan* or *eno*-ΔH2 mutant strains, the upstream and downstream regions of the targeted sequences were amplified via PCR using specific primers. Subsequently, fusion PCR was conducted, and the resulting products were cloned into the plasmid pSET4s. Detailed information regarding the primers can be found in Additional file 8. The constructs were introduced into SS2 competent cells through electroporation, and mutant isolation was achieved following a previously established protocol [20]. The *eno*::*kan* and *eno*-ΔH2 mutant alleles were verified by PCR and sequencing.

### Expression plasmids

Full length *eno* gene from the 05ZYH33 strain was synthesized by Tsingke Biotechnology, which was cloned into pEGFP-C1. Subsequently, the sequences encoding Eno-WT, Eno-ΔN-domain, Eno-ΔC-domain were amplified using PCR with specific primers, as outlined in Additional file 8. The purified fragments were ligated into pET-28a or pcDNA3.1–3×HA using T4 DNA ligase (TaKaRa, 2011 A). Mutations (ΔS, ΔH1, ΔH2, ΔH3 and ΔH4) were introduced through mutagenesis PCR, employing primers detailed in Additional file 8. CFP-Parkin, pCMV-LONP1-3×HA-Neo and pCMV-HSP90AA1-3×FLAG-Neo were purchased from Miaoling Bio.

### Construction of stable hBMEC cell lines overexpressing EGFP-MTS or CLDN5 and PINK1 knockout hBMEC cell lines

psPAX2 and pMD2G plasmids were co-transfected with pBABE-mCherry-GFP-FIS1^101–152^ (MRC PPU Reagents and Services), pLD-puro-MTS-EGFP (BIO SCI), pLV3-CMV-CLDN5-3×FLAG-CopGFP-Puro (Miaoling Bio) or pLenti-PINK1-sgRNA (Beyotime) into HEK293T cells. After 48 h, the supernatants were collected, subsequently filtered using 0.45 μm filters. The culture medium containing lentivirus was then incubated with hBMECs, and this medium was replaced with fresh medium 24 h post-infection. The infected hBMECs were subsequently subjected to selection with 4 μg/mL puromycin and isolated into single cells via flow cytometry.

### Antibodies

For immunofluorescence or western blot, the following antibodies were applied: Rabbit anti-LC3A/B monoclonal antibodies (Cell Signaling Technology, 12,741, western 1:1000, immunofluorescence 1:200); Rabbit anti-LAMP1 monoclonal antibodies (Abclonal, A16894, immunofluorescence 1:200); Mouse anti-LAMP1 monoclonal antibodies (Beyotime, AG2482, immunofluorescence 1:50); Rabbit anti-TOM20 monoclonal antibodies (Beyotime, AF1717, western 1:1000, immunofluorescence 1:200); Mouse anti-HSP60 monoclonal antibodies (Beyotime, AF0186, western 1:1000, immunofluorescence 1:200); Rabbit anti-CLDN5 polyclonal antibodies (Proteintech, 29767-1-AP, western 1:500, immunofluorescence 1:100, IHC 1:800); Rabbit anti-ZO-1 polyclonal antibodies (Abclonal, A0659, western 1:500); Rabbit anti-occludin polyclonal antibodies (Abclonal, A2601, western 1:500); Rabbit anti-PINK1 polyclonal antibodies (Proteintech, 23274–1-AP, western 1:500, immunofluorescence 1:200); Mouse anti-β-Tubulin monoclonal antibodies (Proteintech, 10068-1-AP, western 1:1000); Rabbit anti-His monoclonal antibodies (Cell Signaling Technology, 12698, western 1:1000); Rabbit anti-HA monoclonal antibodies (Cell Signaling Technology, 3724, western 1:1000); Mouse anti-FLAG monoclonal antibodies (Sigma, F1804, western 1:1000, immunofluorescence 1:400); Mouse anti-Eno polyclonal antibodies(Produced by our lab, western 1:500, immunofluorescence 1:200); HRP-conjugated goat anti-rabbit IgG (H + L) secondary antibodies (Beyotime, A0208, western 1:1000); HRP-conjugated goat anti-mouse IgG (H + L) secondary antibodies (Beyotime, A0216, western 1:1000); YSFluor™ 488 goat anti-rabbit IgG (H + L) secondary antibodies (Yeasen, 33106ES60, immunofluorescence 1:200); YSFluor™ 594 goat anti-mouse IgG (H + L) secondary antibodies (Yeasen, 33212ES60, immunofluorescence 1:200); YSFluor™ 647 goat anti-mouse IgG (H + L) secondary antibodies (Yeasen, 33213ES60, immunofluorescence 1:200).

### SS2 invasion assay

1×10^6^ hBMECs cultured in 12-well plates were challenged with SS2 suspended in DMEM at a multiplicity of infection (MOI) of 100. Subsequently, the plates were centrifuged at 800 *g* for 5 min. For western blot analysis, samples were collected at various time points. For immunofluorescence staining, samples were fixed after 4 h of invasion. To quantify bacterial intracellular survival, cells infected with SS2 for 1 h were further incubated with fresh culture medium containing 100 µg/mL gentamicin and 100 U/mL penicillin for an additional 3 h. Subsequent to lysis with 1% saponin (Macklin, S817818), the cell lysates underwent serial dilution and were plated on Todd Hewitt Plus Yeast Extract (THY) agar. The plates were incubated at 37 °C for 36 h to facilitate the enumeration of colony-forming units (CFU).

### Survival assays of SS2 in whole blood

Fresh whole blood was obtained from healthy pigs. SS2 was cultured until reaching the logarithmic growth stage, after which the cells were harvested, washed, and adjusted in PBS to an OD_600_ of 0.2. For the ex vivo survival assay, 100 μL of the prepared bacterial suspension was added to 1 mL of porcine blood. The samples were maintained at 37 °C and gently mixed at intervals during incubation. Bacterial persistence in blood was assessed at the beginning of the assay and after 1 h and 3 h. The bacterial count obtained immediately after inoculation was designated as the baseline and normalized to 100%, and survival at later time points was expressed relative to this initial value.

### Protein purification

*Escherichia coli* BL21(DE3) transformed with constructed pET28a vectors ligating with full-length *eno* gene or its variants were cultured in Luria–Bertani broth at 37 °C with the presence of 50 μg/mL kanamycin. To induce recombinant protein expression, 1 mM IPTG (aladdin, l104812) was added, and then bacteria were grown at 16 °C for 12 h. Post-induction, the bacterial cells were collected via centrifugation and lysed by an ultrasonic cell crusher. The cell lysates were centrifuged at 12 000 *g* for 30 min, and the supernatants were applied for protein purification by Ni-TED sepharose (Sangon Biotech, C600033) following instruction from manufacturer.

### Western blot

Cells were lysed using RIPA lysis buffer (Beyotime, P0013B) and subsequently boiled in loading buffer for 5 min prior to separation by SDS–polyacrylamide gel electrophoresis (SDS-PAGE). The separated proteins were transferred onto PVDF membranes (Bio-Rad, 1620177) at a constant current of 300 mA for 90 min. Afterwards, the membranes were adequately blocked with 5% bovine serum albumin for 2 h at room temperature. The primary antibodies diluted in a specific antibody dilution buffer (Beyotime, P0023A) incubated with membranes at 4 °C for 12 h. Membranes were then incubated with horseradish peroxidase (HRP)-conjugated secondary antibodies at room temperature for 2 h. Finally, the HRP activity was detected using Immobilon Western Substrate (Millipore, WBKLS).

### Immunofluorescence

Cells seeded on glass coverslips were fixed with 4% paraformaldehyde (Biosharp, BL539A) for 20 min at room temperature. Cells were permeabilized and blocked with QuickBlock buffer (Beyotime, P0260) for 30 min with shaking, which were then incubated with primary antibodies diluted in immunofluorescent primary antibody dilution buffer (Beyotime, P0262) at 4 °C overnight. Subsequently, slides were incubated with fluorophore-conjugated secondary antibodies diluted in immunofluorescent secondary antibody dilution buffer (Beyotime, P0265) at room temperature for 2 h. Finally, nuclei were stained with 10 µg/mL DAPI (Solarbio, C0065) for 10 min at room temperature. Fluorescent images were captured using a Nikon confocal microscope.

### Transmission electron microscopy

The cells were fixed with 2.5% glutaraldehyde at 4 °C overnight, followed by incubation with 1% osmium tetroxide for 1 h. Subsequently, the samples underwent a one-hour staining process with 2% uranyl acetate, after which they were dehydrated using acetone and embedded in epoxy resin. Sections were stained with uranyl acetate and lead citrate, and visualization was performed using a transmission electron microscope (Hitachi).

### *mito*-QC assay

hBMECs stably expressing mCherry-GFP-FIS1^101–152^ (*mito*-QC reporter) were seed on glass bottom cell culture dishes (NEST, 801002). Following a 4-h invasion with SS2, the cells were fixed and subsequently imaged using a Nikon confocal microscope. Puncta displaying red fluorescence in the absence of green fluorescence were classified as mito-lysosomes and quantified in each cell.

### Flow cytometry

5 × 10^6^ hBMECs were exposed to 2 µg/mL rEno-WT or rEno-ΔH2 for 4 h. TMRM staining was employed to assess MMP. Following protein treatment, hBMECs were incubated with 100 mM TMRM (Thermo Fisher, T668) for 30 min at 37 °C. Apoptosis induced by rEno was evaluated using the Annexin V-FITC Apoptosis Detection Kit (DOJINDO, AD10) in accordance with the manufacturer's instruction. All samples were analyzed using the CytoFLEX flow cytometer (Beckman), and data processing was performed with FlowJo software.

### EGFP-MTS mitochondrial fluorescence import assessment

Stable hBMECs expressing EGFP-MTS were transiently transfected with pCMV-HSP90AA1-3×FLAG-Neo or empty vector, which was selected via the treatment with G418. Afterwards, the cells were stimulated with 2 μg/mL rEno for 4 h, and then 0.1 μM of MitoBright LT Red (DOJINDO, MT11) was incubated with cells for 30 min at 37 °C to label mitochondria. The colocalization between EGFP-MTS and mitochondria was detected by confocal microscope (Nikon), which was quantified by thresholded M2 (tM2) Manders coefficient.

### Co-IP and LC–MS/MS analysis

Cells were lysed with immunoprecipitation lysis buffer (Thermo Fisher, 87787) supplemented with protease inhibitors (Roche, 04693159001) on ice for 20 min. The cell lysates were centrifuged at 12 000 *g* for 25 min, and the supernatants were incubated with anti-HA antibodies-coated agarose (Lablead, HNA-25–500) or anti-His antibodies-coated agarose (Abbkine, A02050AGB) at 4 °C for 2 h. Agarose was washed and collected via centrifuge at 2500 *g*, with finally adherent proteins eluted with 2 × SDS loading buffer. Samples were boiled at 95 °C for 10 min, which underwent western blot to detect target proteins.

To identify proteins interacting with Eno, only pcDNA-3.1–3×HA-Eno was transfected, and HA-Eno was concentrated with anti-HA antibodies-coated agarose. Samples were delivered to Scientific Research Center of Wenzhou Medical University for further processing and identification. Briefly, samples compressed by SDS-PAGE were chemically treated (reduced with TCEP and alkylated with IAA) to prepare proteins for digestion. Next, trypsin enzyme was added to digest the proteins into peptides. Subsequently, the peptides were extracted using an acidified acetonitrile solution, desalted, and concentrated. Finally, peptides were analyzed using EASY-nLC 1200 coupled to Orbitrap Fusion Lumos for identification.

### Pull-down assay

Purified His-tagged rEno-WT or rEno-ΔH2 was first immobilized on Ni–NTA agarose beads at 4 °C for 1 h with gentle rotation. After washing with binding buffer to remove unbound rEno proteins, the rEno-loaded beads were incubated with purified Flag-rHSP90AA1 (OriGene, TP312496) at 4 °C for 2 h. The beads were then collected by centrifugation and washed five times with binding buffer to remove nonspecifically associated proteins. Bound protein complexes were eluted using imidazole-containing elution buffer and analyzed by SDS-PAGE and western blotting.

### LDH assay

1×10^6^ hBMECs seeded in 12-well cell culture plates were subjected to the treatment of rEno-WT or rEno-ΔH2 at 2 µg/mL for 4 h or 6 h. Following this, the cell culture media was collected and the concentration of LDH was evaluated via the Lactate Dehydrogenase Assay Kit (Nanjing Jiancheng Bioengineering Institute, A020-2-2).

### In vitro BBB model construction and EB permeation assay

Collagen type I (Sigma, C7774) was used to coat Transwell chambers (1.12 cm^2^, 3 µm pore size), followed by overnight drying. 5 × 10^4^ hBMECs were then seeded onto the apical surface, which were cultured for 7–10 days to establish confluent monolayers. To assess the effect of Eno on barrier integrity, monolayers with or without Mdivi-1 pretreatment were incubated with 2 µg/mL rEno. The inserts were subsequently relocated to new plates with Hank's Balanced Salt Solution (HBSS) in the lower chamber. 50 µL of 0.4% EB, dissolved in PBS, was introduced into the upper chamber. The assembly was then incubated at 37 °C with 5% CO₂ for a duration of 40 min. The permeability of monolayers was assessed colorimetrically by measuring the optical density at 600 nm (OD₆₀₀) in the lower chamber.

### Ethics statement and animal experiments

All animal experiments were approved by the Laboratory Animal Center and Ethics Committee of Wenzhou Medical University (Approval No. wydw2025-0166) and strictly followed established welfare standards.

Five-week-old female C57BL/6 J mice (sourced from Zhejiang Vital River Laboratory Animal Technology Co. Ltd.), housed under specific pathogen-free (SPF) conditions, were randomly assigned using a computer-generated randomization sequence into six groups (*n *= 6 per group, chosen based on previous similar studies). Mice were intraperitoneally inoculated with 5 × 10⁸ CFU of the WT strain or the mutant strain, which were then treated with or without Mdivi-1 (50 mg/kg per day) 2 h post-infection. 48 h post-infection, brain samples were aseptically collected and homogenized in PBS. The homogenates underwent serial dilution and were subsequently plated on THY agar plates. The plates were incubated at 37 °C for 48 h, after which CFU were quantified. For BBB permeability assessment, mice received an intravenous injection of 100 µL of 2% EB after 2 days of infection. Two hours later, EB was extracted from the brains using formamide, and then the concentration of EB was determined by measuring absorbance at 620 nm (OD₆₂₀). For immunohistochemistry (IHC), brain tissues were subjected to fixation in 4% paraformaldehyde (Biosharp, BL539A) for 48 h prior to subsequent processing. Outcome assessors were blinded during EB extravasation measurements and IHC evaluations.

### Immunohistochemistry

The fixed brain tissues were embedded in paraffin and sectioned at a thickness of 4 μm. The sections were stained utilizing the SABC kit (BOSTER, SA1022) in accordance with the manufacturer's protocol, followed by incubation with rabbit anti-CLDN5 antibodies at 4 °C for 12 h. Visualization was subsequently achieved using the DAB colorimetric kit (BOSTER, AR1027). The integrated optical density of each stained area (IOD/area) was quantitatively analyzed.

### Quantification and statistics

All experiments included a minimum of three biological replicates. Confocal microscopy image quantification analyzed at least 50 cells per group. The normality of the datasets was evaluated using the Shapiro–Wilk test, and statistically significant differences were analyzed using one-way ANOVA, two-way ANOVA, t-test, Mann–Whitney test or Kruskal–Wallis test in GraphPad Prism 9. A *P* < 0.05 was considered to indicate a statistically significant difference.

## Results

### SS2 infection induces mitophagy in hBMECs

**Figure 1:**
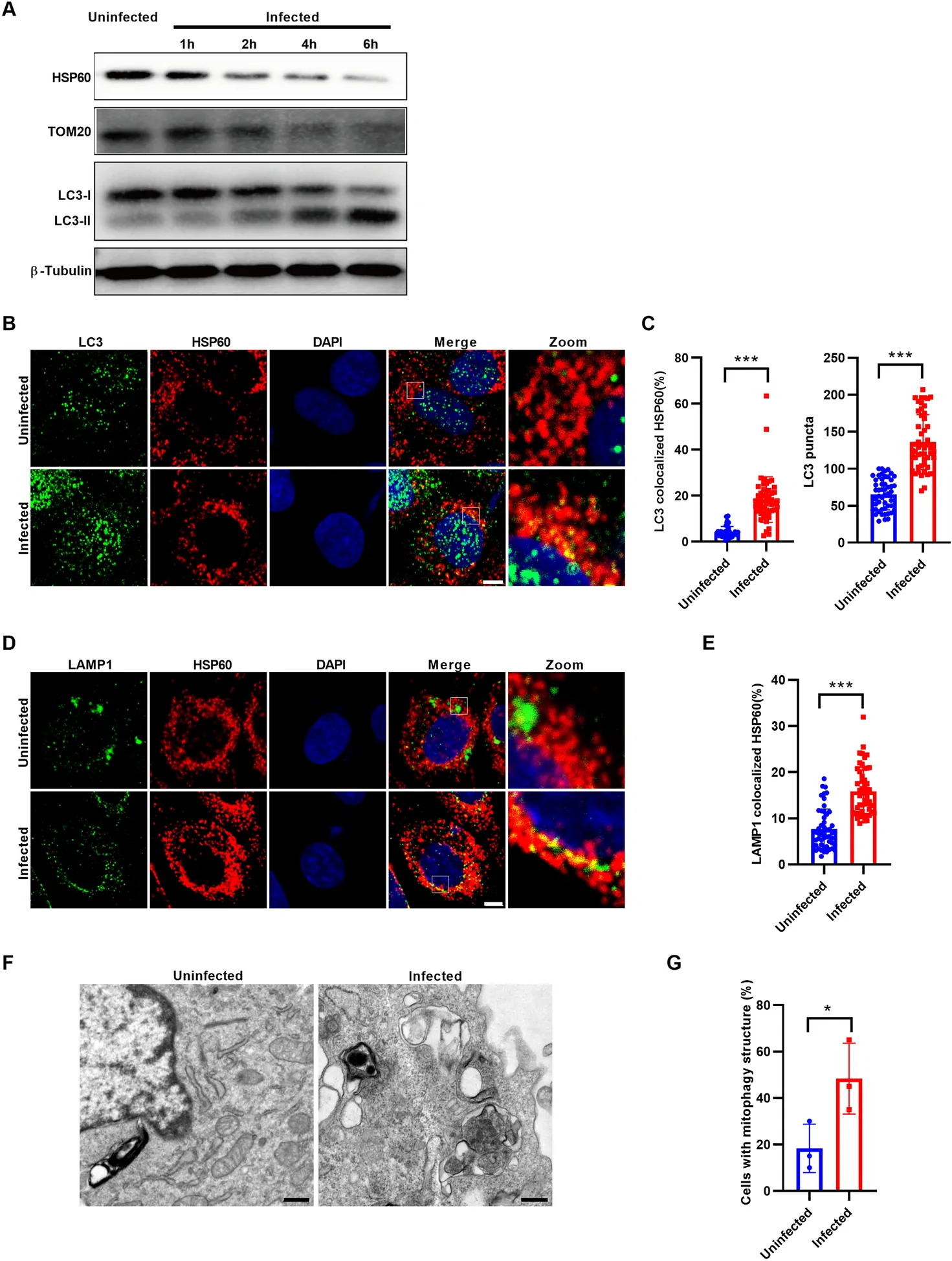
**SS2 infection induces mitophagy in hBMECs.** **A** Western blot analysis of LC3, HSP60, and TOM20 protein levels in hBMECs at 1, 2, 4, and 6 h post-SS2 infection. Uninfected cells served as control. β-Tubulin was used as loading control. **B** Confocal images of LC3 and HSP60 colocalization in infected and uninfected hBMECs. Nuclei were stained with DAPI. Scale bar: 5 µm. **C** Quantification of the percentage of HSP60 colocalized with LC3 and the number of LC3 puncta per cell in panel B. Data are mean ± s.d.; *n *= 51 cells from three independent experiments. Significant differences were identified using Mann–Whitney test. ***, *P* < 0.001. **D** Confocal microscopy images of LAMP1 and HSP60 colocalization in SS2-infected and uninfected hBMECs. Nuclei were stained with DAPI. Scale bar: 5 µm. **E** Quantification of the percentage of HSP60 colocalized with LAMP1 in panel D. Data are mean ± s.d.; *n *= 51 cells from three independent experiments. Significant difference was identified using Mann–Whitney test. ***, *P* < 0.001. **F** Representative TEM images of mitophagy structure (mitochondria enclosed by autophagosome or autolysosome) after SS2 infection. Scale bar: 500 nm. **G** Percentages of cells with mitophagy structure in panel F. 60 cells were quantified in each group from three independent experiments. Data are mean ± s.d.; *n* = 3. Significant difference was identified using an unpaired t test. *, *P* < 0.05.

To investigate whether SS2 can induce mitophagy in hBMECs, cells were collected in a time-series after SS2 invasion. The levels of intracellular autophagy marker protein LC3, mitochondrial matrix protein HSP60 and outer membrane protein TOM20 were determined via western blot analysis. The results indicated a progressive increase in the intensity of LC3-II bands and a corresponding decrease in LC3-I bands with prolonged infection time (Figure 1A). Additionally, the protein levels of TOM20 and HSP60 exhibited a gradual decline, suggesting the induction of mitophagy in hBMECs (Figure 1A). To further substantiate this phenomenon, cells were fixed 4 h post-infection, then LC3 or lysosome marker LAMP1 were co-stained with HSP60 using specific antibodies. As illustrated in Figure 1B and C, there was a significant increase in the number of intracellular LC3 puncta and the proportion of mitochondria colocalized with LC3 following SS2 infection in hBMECs. Similarly, stronger colocalization between LAMP1 and HSP60 in infected cells was observed (Figure 1D and E), confirming that mitochondria were degraded in the lysosome. Transmission electron microscope (TEM) revealed that more mitochondria were enclosed in double membrane structure (autophagosome) or single membrane structure (autolysosome) in infected cells (Figure 1F and G). Collectively, these experimental results proved that SS2 infection effectively induces mitophagy in hBMECs.

### Eno is indispensable for SS2-induced mitophagy

**Figure 2:**
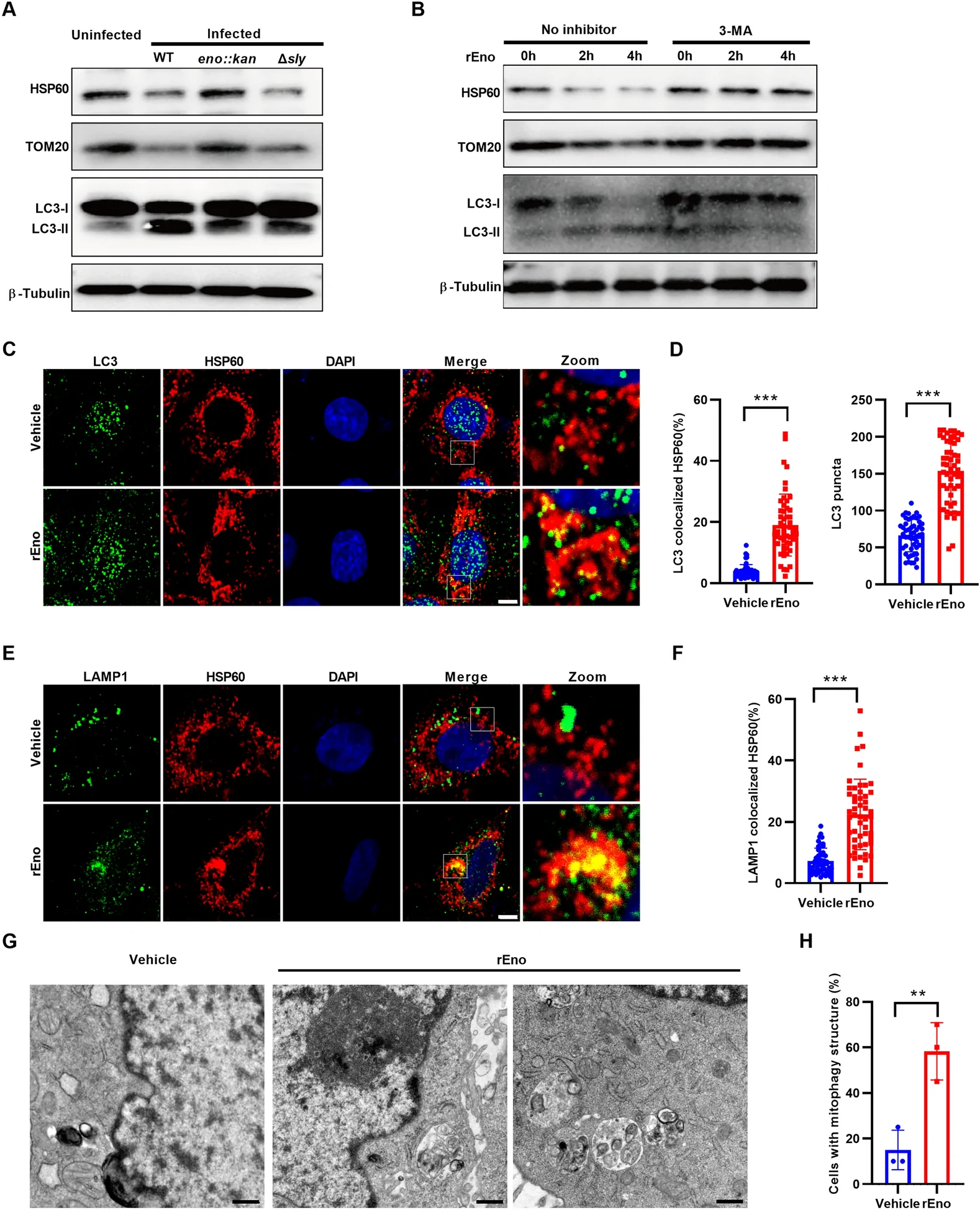
**SS2 induces mitophagy via Eno.** **A** Western blot analysis of HSP60, TOM20, and LC3 in hBMECs infected with wild-type (WT), *eno::kan*, or Δ*sly* strains. Uninfected cells served as control. β-Tubulin was used as loading control. **B** Western blot analysis of HSP60, TOM20, and LC3 in hBMECs after 2 µg/mL rEno treatment, with or without 3-MA pretreatment. β-Tubulin was used as loading control. **C** Confocal images of LC3 and HSP60 colocalization in vehicle- or rEno-treated hBMECs. Nuclei were stained with DAPI. Scale bar: 5 µm. **D** Quantification of the percentage of HSP60 colocalized with LC3 and the number of LC3 puncta per cell in panel C. Data are mean ± s.d.; *n *= 51 cells from three independent experiments. Significant differences were identified using Mann–Whitney test. ***, *P* < 0.001. **E** Representative confocal microscopy images of colocalization between LAMP1 and HSP60 in hBMECs treated with vehicle or rEno. Nuclei were stained with DAPI. Scale bar: 5 μm. **F** Quantification of the percentage of HSP60 colocalized with LAMP1 in panel E. Data are mean ± s.d.; *n *= 51 cells from three independent experiments. Significant difference was identified using Mann–Whitney test. ***, *P* < 0.001. **G** Representative TEM images of mitophagy structure in rEno-treated hBMECs. Scale bar: 500 nm. **H** Percentages of cells with mitophagy structure in panel G. 60 cells were quantified in each group from three independent experiments. Data are mean ± s.d.; *n *= 3. Significant difference was identified using an unpaired t test. **, *P* < 0.01.

Given that pathogen-mediated mitophagy frequently depends on virulence proteins entering into host cells, our study concentrated on the SS2 proteins known to be released extracellularly. SS2 suilysin (SLY) has been reported to induce autophagy in microglia [21], while enolase (Eno), detectable in the extracellular space, is linked to mitochondrial damage [22, 23]. This suggests their potential roles in the SS2-mediated induction of mitophagy. To assess this hypothesis, we generated the *sly* deletion mutant strain (Δ*sly*). However, because a complete deletion of *eno* could not be obtained, we partially disrupted *eno* by inserting a kanamycin-resistance cassette at its coding sequence, thereby generating the *eno*::*kan* mutant. Western blot analysis using an anti-Eno antibody showed that, compared with the WT strain, the *eno*::*kan* mutant strain displayed no obvious band at the expected Eno position (Additional file 1). Importantly, no significant differences in growth kinetics, survival in whole blood or cellular invasion efficiency were observed between the *eno*::*kan* mutant and the WT strain (Additional file 1). The conversion of LC3-I to LC3-II, typically induced by infection, was markedly reduced in cells infected with both the *eno::kan* and Δ*sly* mutant strains compared to the wild-type strain (WT), indicating that both Eno and SLY are capable of activating autophagic flux (Figure 2A). However, the degradation of HSP60 and TOM20 was fully abolished following the *eno::kan* mutant strain invasion in contrast to cells infected with the WT strain, whereas the knockout of *sly* had a negligible impact on the levels of these two mitochondrial marker proteins (Figure 2A). *mito*-QC analysis revealed that infection with the WT strain led to the increased formation of mito-lysosomes in comparison to uninfected cells, but this process was entirely inhibited following the disruption of the *eno* (Additional file 2). Significantly, following the infection of hBMECs with SS2, immunodetection utilizing anti-Eno antibodies demonstrated that Eno, in addition to its presence within the bacterial cell, was capable of entering the host cytoplasm and partially surrounding or engaging with mitochondria (Additional file 3), which providing spatial feasibility for its induction of mitophagy. These results suggested that Eno, rather than SLY, plays a critical role in mitophagy induction.

To further investigate the ability of Eno to induce mitophagy, we treated hBMECs with recombinant Eno protein (rEno) expressed in *E. coli*. Western blot analysis revealed that 2 µg/mL rEno significantly facilitated the conversion of LC3-I to LC3-II and promoted the degradation of HSP60 and TOM20 (Figure 2B). This effect was progressively enhanced with prolonged rEno exposure and was completely inhibited by the autophagy inhibitor 3-methyladenine (3-MA) (Figure 2B). Furthermore, confocal microscopy images demonstrated a significant increase in the number of LC3 puncta within cells and a higher proportion of mitochondria colocalized with LC3 or LAMP1 following rEno treatment for 4 h (Figure 2C–F). Under TEM, it was obvious that rEno caused the change of mitochondrial morphology, including disappearance of cristae. Importantly, more formation of autophagosomal and autolysosomal capturing mitochondria structures was observable (Figure 2G and H). Altogether, these multi-dimensional findings suggested that the Eno from SS2 specifically activates mitophagy in hBMECs.

### Eno activates PINK1-Parkin dependent mitophagy

**Figure 3:**
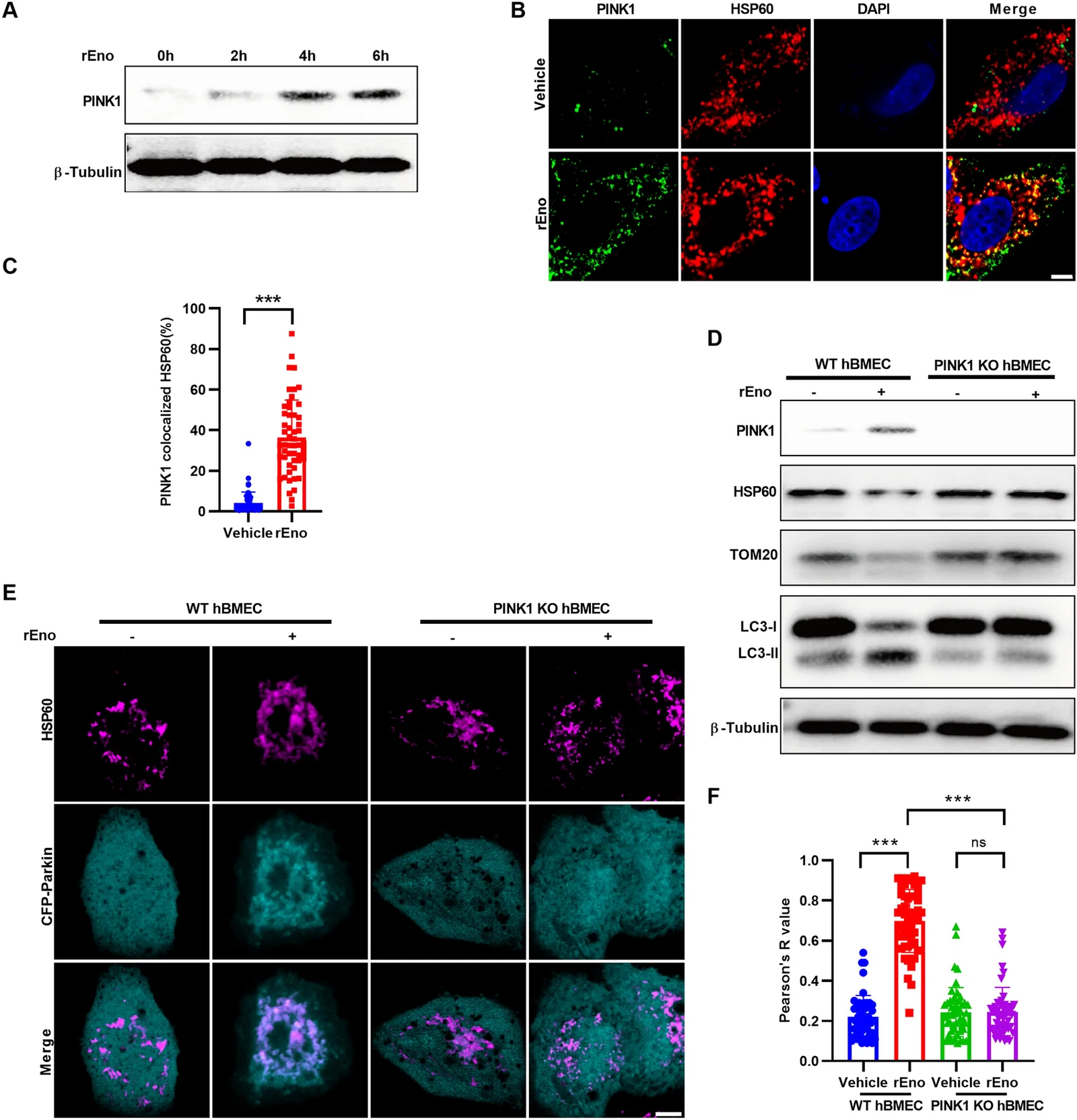
**Eno activates mitophagy through the PINK1-Parkin pathway.** **A** Western blot analysis of PINK1 in hBMECs treated with rEno for 0, 2, 4, and 6 h. β-Tubulin was used as loading control. **B** Representative confocal microscopy images of colocalization between PINK1 and HSP60 in hBMECs treated with vehicle or rEno. Nuclei were stained with DAPI. Scale bar: 5 μm. **C** Quantification of the percentage of HSP60 colocalized with PINK1. Data are mean ± s.d.; *n *= 53 cells from three independent experiments. Significant difference was identified using Mann–Whitney test. ***, *P* < 0.001. **D** Western blot analysis of PINK1, HSP60, TOM20, and LC3 in wild-type (WT) and PINK1 knockout (PINK1 KO) hBMECs, with or without rEno treatment. β-Tubulin served as the loading control. **E** Confocal images of CFP-Parkin and HSP60 in WT or PINK1 KO hBMECs post-rEno treatment. Scale bar: 5 µm. **F** Pearson’s correlation coefficient analysis of CFP-Parkin and HSP60 colocalization from panel E. Data are mean ± s.d.; *n *= 51 cells from three independent experiments. Significant differences were identified using Kruskal–Wallis test. ***, *P* < 0.001.

The activation of the PINK1-Parkin pathway is recognized as an important molecular mechanism for inducing mitophagy [24, 25]. Under normal physiological conditions, PINK1 is rapidly degraded. However, upon mitochondrial damage, PINK1 accumulates within the mitochondria, subsequently activating and recruiting Parkin. To investigate the role of the PINK1-Parkin pathway in mitophagy induction by Eno, we primarily examined the dynamic changes in PINK1 protein levels in hBMECs following rEno stimulation. Addition of rEno led to a significant increase in intracellular PINK1 protein levels in a time dependent manner (Figure 3A). Further subcellular localization analysis revealed that rEno markedly enhanced PINK1 accumulation on mitochondria, significantly increasing the proportion of mitochondria colocalized with PINK1 (Figure 3B and C). The knockout of PINK1 in hBMECs completely inhibited the mitophagy induced by rEno (Figure 3D). Following the overexpression of CFP-Parkin in hBMECs and subsequent stimulation with rEno for 4 h, a strong colocalization of CFP-Parkin and HSP60 was observed (Figure 3E and F). As expected, this colocalization was entirely abolished by the knockout of PINK1 (Figure 3E and F). These findings indicated that the PINK1-Parkin pathway plays a crucial role in the Eno-induced mitophagy.

### Eno interacts with HSP90AA1 to induce mitophagy

**Figure 4:**
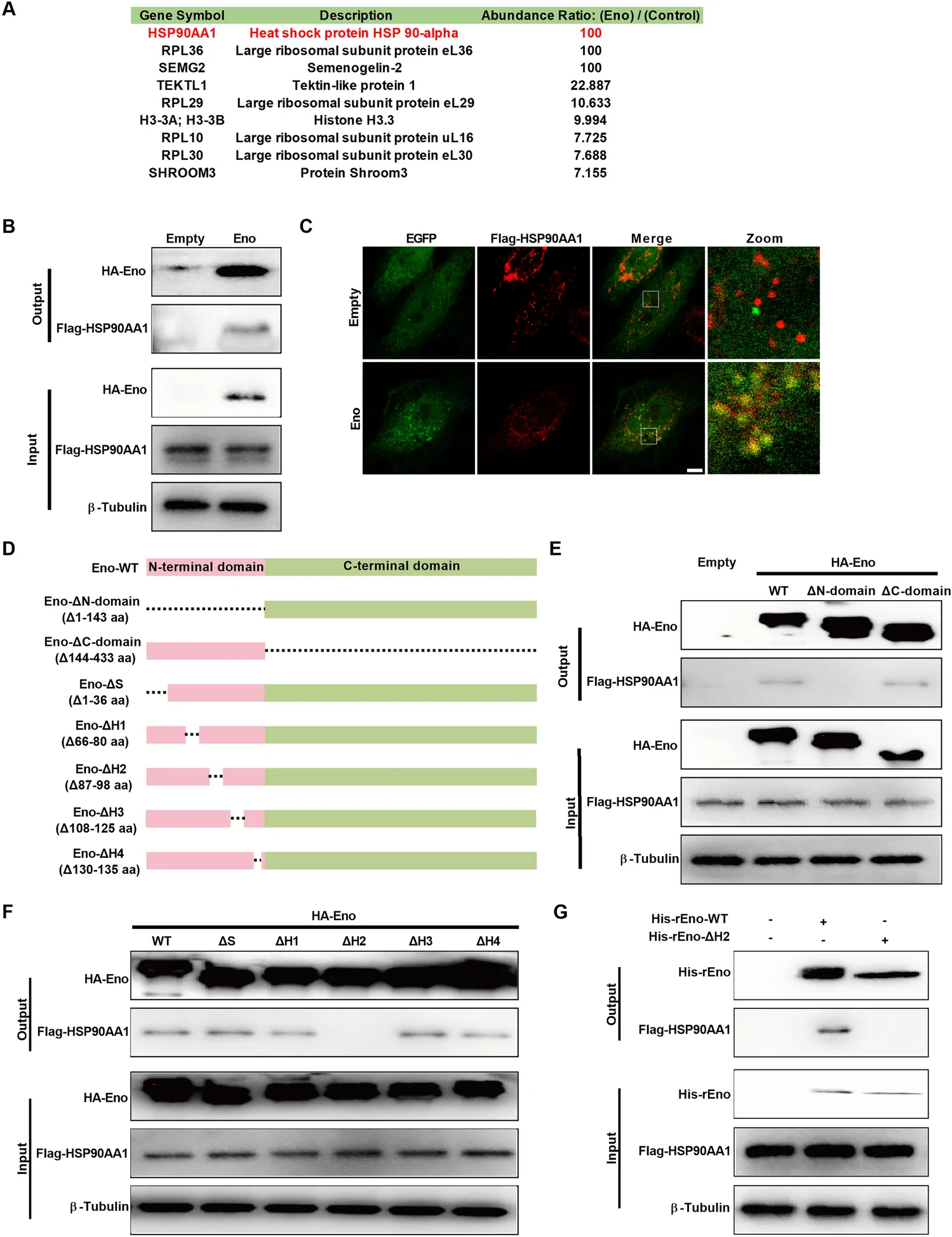
**Eno specifically interacts with HSP90AA1.** **A** Mass spectrometry analysis of proteins co-immunoprecipitated with HA-Eno from hBMEC lysates. Top panel shows protein candidates with their abundance ratios (Eno/Control). **B** Co-IP validation of the specific interaction between HA-Eno and FLAG-HSP90AA1 by western blot. hBMECs transfected with empty vector (empty) were used as blank control. β-Tubulin served as loading control for input lysates. **C** Confocal microscopy images of hBMECs expressing EGFP-Eno or EGFP, immunostained for FLAG-HSP90AA1. Yellow signals in merged images indicate colocalization. Scale bar: 5 µm. **D** Diagram of wild type Eno and its truncations. **E** Co-IP analysis of FLAG-HSP90AA1 interacting with full-length (WT), N-terminal domain-deleted (ΔN-domain), or C-terminal-domain-deleted (ΔC-domain) Eno. hBMECs transfected with empty vector (empty) were used as blank control. β-Tubulin served as loading control for input lysates. **F** Identification of the interaction between FLAG-HSP90AA1 and HA-Eno variants (WT: full-length; ΔS: all β-sheet deletion; ΔH1~ΔH4: α-helix deletions) via Co-IP analysis. hBMECs transfected with empty vector (empty) were used as blank control. β-Tubulin served as loading control for input lysates. **G** Western blot analysis showing co-immunoprecipitation of FLAG-HSP90AA1 with rEno-WT and rEno-ΔH2. β-Tubulin served as loading control for input lysates.

To conduct an in-depth analysis of the molecular mechanism of mitophagy induced by Eno, HA-tagged Eno (HA-Eno) was ectopically expressed in hBMECs. Following enrichment using antibodies-coated beads, mass spectrometry was employed to identify host proteins interacting with HA-Eno. The analysis revealed that HSP90AA1 was specifically present in the HA-Eno transfected group, while it was absent in the group transfected with empty vector (Figure 4A). To verify the specificity of this interaction, Co-immunoprecipitation (Co-IP) combined with western blot analysis demonstrated that FLAG-HSP90AA1 exhibited a co-immunoprecipitation signal exclusively in HA-Eno enriched samples, with no signal detected in the negative control samples (Figure 4B). Under confocal microscope FLAG-HSP90AA1 showed strong colocalization with EGFP-Eno, but not with EGFP (Figure 4C), which further confirmed the interaction of Eno with HSP90AA1. Considering that the Eno of SS2 comprises an N-terminal domain and a C-terminal domain (Figure 4D), we constructed expression vectors for the N-terminal domain deletion mutant (HA-Eno-ΔN-domain) and the C-terminal domain deletion mutant (HA-Eno-ΔC-domain) to identify the key interaction domain. Interestingly, HA-Eno-ΔN-domain completely lost its ability to interact with HSP90AA1, whereas HA-Eno-ΔC-domain retained normal binding activity (Figure 4E), indicating that the N-terminal domain of Eno is critical for its interaction with HSP90AA1. Subsequent structural analysis identified that the N-terminal domain comprises three inverted β-sheets and four cross-linked α-helices (Figure 4D). To pinpoint the specific amino acid sequence essential for this interaction, we generated five variants of Eno: one lacking all β-sheets (Eno-ΔS) and the remaining four each lacking a single α-helix (Eno-ΔH1~H4), and co-expressed them with FLAG-HSP90AA1 in hBMECs. The variant lacking the second α-helix (Eno-ΔH2, residues 87–98) was unable to interact with HSP90AA1, whereas mutations in the remaining amino acid sequences did not disrupt the interaction (Figure 4F). To replicate the actual infection state, hBMECs overexpressing FLAG-HSP90AA1 were incubated with rEno-WT and rEno-ΔH2 for 4 h, and the cell lysates were immunoprecipitated with anti-His antibodies. Further western blot analysis confirmed that the deletion of H2 resulted in rEno losing its capacity to interact with FLAG-HSP90AA1 (Figure 4G). A pull-down assay was performed using purified rEno-WT, rEno-ΔH2, and recombinant FLAG-rHSP90AA1. FLAG-rHSP90AA1 was efficiently pulled down by rEno-WT, whereas this interaction was abolished with rEno-ΔH2, further demonstrating a direct interaction between Eno and HSP90AA1 that depends on the integrity of the H2 region (Additional file 4). These findings indicated that Eno specifically interacts with HSP90AA1 through its N-terminal domain, and this interaction is critically dependent on the integrity of the second α-helix.

**Figure 5:**
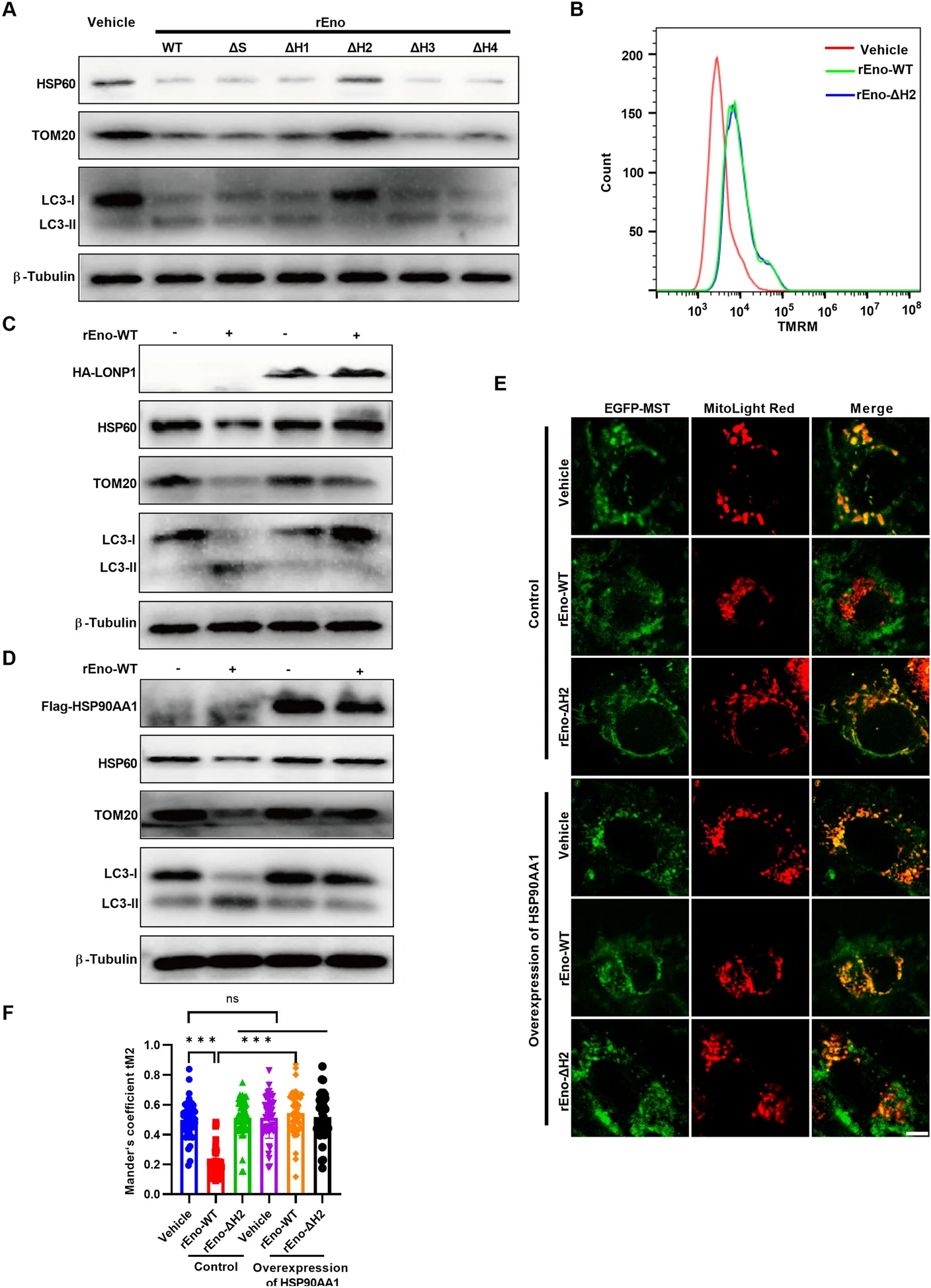
**Eno-HSP90AA1 interaction results in mitochondrial accumulation of misfolded proteins to activate mitophagy.** **A** Western blot analysis of HSP60, TOM20, and LC3 in hBMECs treated with vehicle, full-length rEno (rEno-WT), β-sheet deleted mutant (rEno-ΔS), or α-helix-deleted mutants (rEno-ΔH1~ΔH4). β-Tubulin served as loading control. **B** Flow cytometry analysis of TMRM intensity in hBMECs treated with vehicle, rEno-WT or rEno-ΔH2. **C** Western blot analysis of HSP60, TOM20, and LC3 in hBMECs treated with rEno-WT or vehicle, with or without HA-LONP1 overexpression. β-Tubulin served as loading control. **D** Western blot of HSP60, TOM20 and LC3 in hBMECs treated with rEno-WT or vehicle, with or without FLAG-HSP90AA1 overexpression. β-Tubulin served as loading control. **E** Representative confocal images showing mitochondrial localization of EGFP-MTS in hBMECs treated with vehicle, rEno-WT or rEno-ΔH2, with or without the overexpression of FLAG-HSP90AA1. Mitochondria were stained by MitoLight Red. Scale bar: 5 µm. **F** Thresholded M2 (tM2) Manders coefficient analysis of EGFP-MTS and MitoLight Red colocalization from panel E. Data are mean ± s.d.; *n *= 50 cells from three independent experiments. Significant differences were identified using Kruskal–Wallis test. ***, *P* < 0.001.

To elucidate the impact of the interaction between Eno and HSP90AA1 on mitophagy, rEno-ΔS and rEno-ΔH1~ΔH4 were expressed and purified. rEno-induced mitophagy was fully inhibited upon H2 deletion, but other variants (rEno-ΔS, ΔH1, ΔH3, ΔH4) retained mitophagy-inducing activity comparable to that of the rEno-WT (Figure 5A). MMP loss is a well-established mechanism for initiating mitophagy [26], and therefore we hypothesized that HSP90AA1-Eno interaction reduced MMP. TMRM fluorescence staining showed that rEno-WT dissipated the MMP in hBMECs, whereas the effect of rEno-ΔH2 on MMP did not significantly differ from that of the rEno-WT (Figure 5B). This paradoxical observation suggested that, while the deletion of H2 entirely impeded the mitophagy-inducing function of Eno, its influence on MMP persisted autonomously. The findings indicated that the MMP dissipation is not the primary factor for Eno-induced mitophagy, and other critical mechanisms are involved.

While MMP dissipation triggers PINK1-Parkin-mediated mitophagy, the accumulation of misfolded proteins within the mitochondria can also initiate this process [27]. LONP1, a mitochondrial ion peptidase, plays a role in degrading denatured proteins, which is involved in misfolded proteins induced mitophagy. The overexpression of LONP1 almost entirely inhibited Eno-induced mitophagy, suggesting that the aggregation of misfolded proteins in mitochondria may contribute to Eno-induced mitophagy (Figure 5C). Thus, we hypothesized that the interaction between Eno and HSP90AA1 impairs the function of HSP90AA1, leading to the accumulation of misfolded proteins within the mitochondria, which was supported by the observation that overexpression of HSP90AA1 can also mitigate the mitophagy induced by rEno-WT (Figure 5D). The mitochondrial misfolded proteins can be sensed by protein import motor complex to reduce protein transport [28]. The Mitochondrial Targeting Sequence (MTS)-EGFP mitochondrial transport assay was applied to monitor mitochondrial protein transport [29]. Our results showed that rEno-WT reduced EGFP-MTS mitochondrial transport, which was almost completely restored upon FLAG-HSP90AA1 overexpression (Figure 5E and F). However, the colocalization between EGFP-MTS and mitochondria in rEno-ΔH2-treated cells did not differ from that observed in vehicle-treated cells (Figure 5E, F). Taken together, these data indicated that the accumulation of misfolded proteins in mitochondria, potentially driven by the interaction between Eno and HSP90AA1, contributes to mitophagy induction.

### Eno-induced mitophagy disrupts the BBB via the degradation of CLDN5

**Figure 6:**
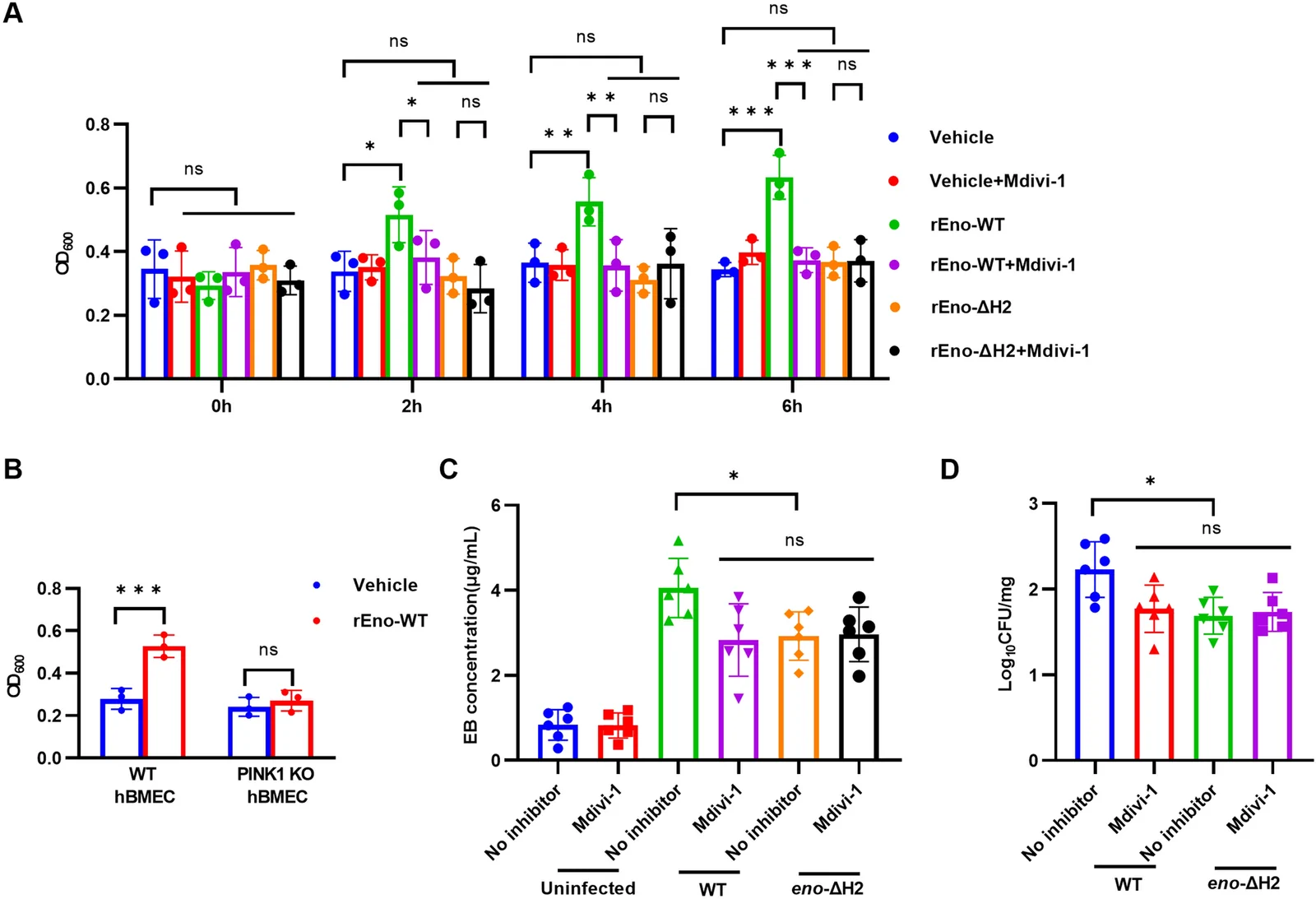
**Eno-induced mitophagy disrupts the integrity of BBB.** **A** EB permeation assay in hBMEC monolayers treated with rEno-WT or rENO-ΔH2, with or without Mdivi-1 pretreatment. Data are mean ± s.d.; *n *= 3. Significant differences were identified using a two-way ANOVA followed by Dunnett’s multiple comparisons test. *, *P* < 0.05, **, *P* < 0.01, ***, *P* < 0.001. **B** EB permeation assay in hBMEC monolayers treated with rEno-WT or vehicle, in the presence and absence of PINK1 knockout. Data are mean ± s.d.; *n *= 3. Significant differences were identified using a two-way ANOVA followed by Dunnett’s multiple comparisons test. ***, *P* < 0.001. **C** The quantification of EB penetrated into brains of C57 mice infected with the WT strain or the *eno*-ΔH2 mutant strain, with or without Mdivi-1 treatment. Data are mean ± s.d.; *n *= 6. Significant differences were identified using a one-way ANOVA followed by Dunnett’s multiple comparisons test. *, *P* < 0.05. **D** The bacterial load in the brains of C57 mice infected with the WT strain or the *eno*-ΔH2 mutant strain, with or without Mdivi-1 treatment. Data are mean ± s.d.; *n *= 6. Significant differences were identified using a one-way ANOVA followed by Dunnett’s multiple comparisons test. *, *P* < 0.05.

Mitophagy induced by bacterial pathogens can enhance their intracellular survival [17], but inhibition of mitophagy via Mdivi-1 showed no impact on SS2 survival in hBMECs (Additional file 5). We next asked whether Eno-induced mitophagy affected the integrity of BBB. To address this question, hBMECs pretreated with or without mitophagy inhibitor Mdivi-1 were exposed to 2 μg/mL rEno-WT or rEno-ΔH2, and the permeability of monolayer cell barrier was analyzed by Evans Blue (EB) permeation experiment. The monolayer barrier permeability was obviously increased by the addition of rEno-WT, while pretreatment with Mdivi-1 effectively alleviated the BBB damage caused by rEno-WT (Figure 6A). Consistently, undifferentiated permeation of EB in the presence of rEno-ΔH2 compared with untreated cells indicated that Eno-induced mitophagy involved in the disruption of the BBB (Figure 6A). Moreover, the knockout of PINK1 nearly completely inhibited the rEno-induced increase in EB permeability (Figure 6B). This evidence further corroborates the adverse effect of rEno-induced mitophagy on the structural integrity of the BBB. Previous studies have reported that Eno can increase the permeability of BBB by inducing apoptosis [12]. But it was worth noting that 2 μg/mL rEno-WT or rEno-ΔH2 did not show cytotoxicity or cause apoptosis through LDH analysis and PI/Annexin V staining (Additional file 6). To further verify whether Eno-induced mitophagy affects permeability of BBB in vivo, mice were challenged with either the WT strain or the mutant strain with the deletion of H2 in Eno (*eno*-ΔH2), a precisely targeted mutant chosen to avoid potential in vivo confounding variables associated with the *eno*::*kan* insertion. In the brains of mice infected with the *eno*-ΔH2 strain, there were significantly decreased levels of detectable EB in comparison to those infected with the WT strain (Figure 6C). Mdivi-1 treatment obviously reduced the amount of EB entering brains in WT strain infected mice to that of *eno*-ΔH2 strain infected mice (Figure 6C). As expected, the application of Mdivi-1 or the deletion of H2 from Eno can decrease the bacterial burdens in brains (Figure 6D). The above results confirmed that Eno-induced mitophagy leads to BBB disruption in SS2 infection.

**Figure 7:**
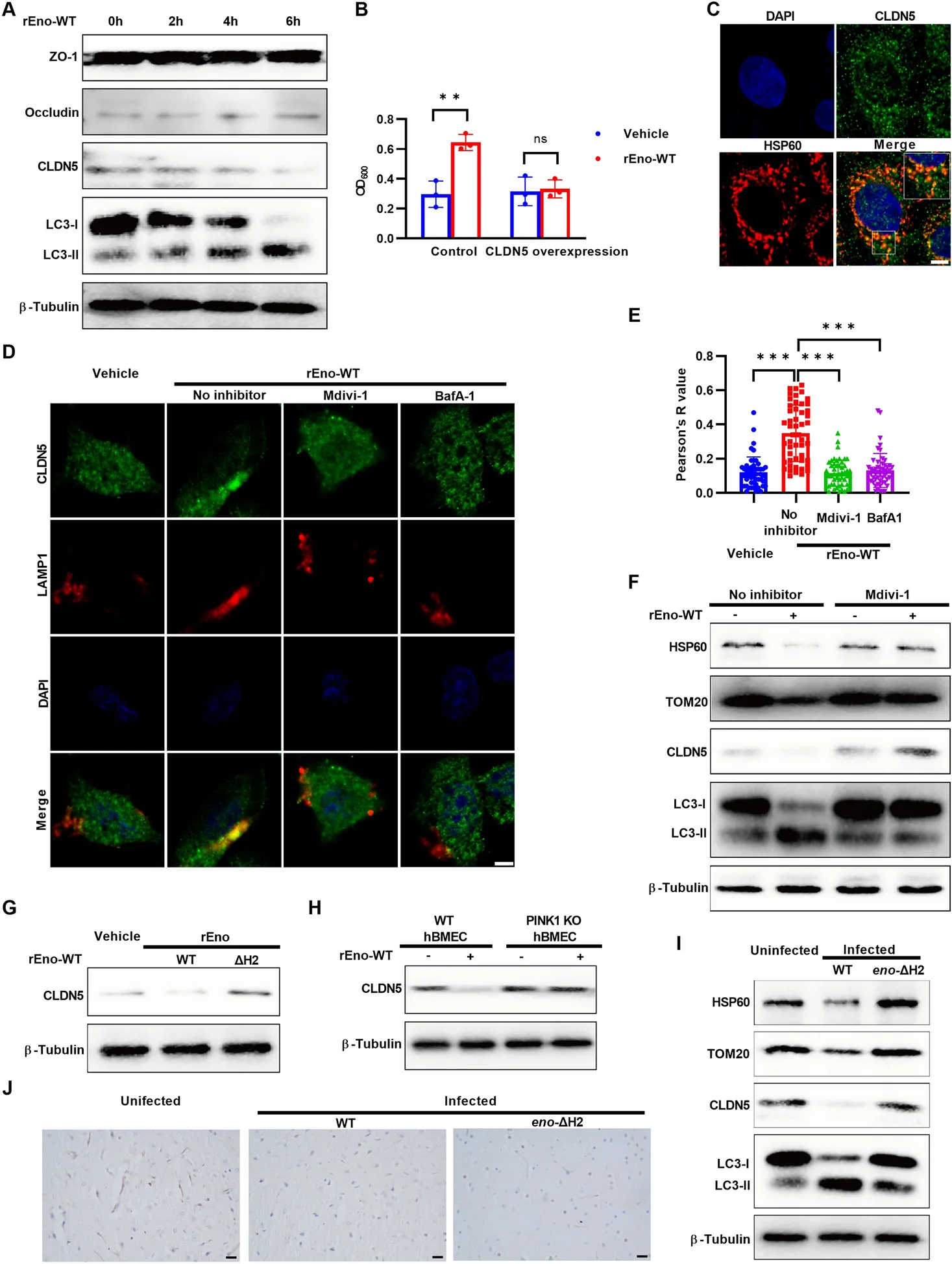
**Eno-mediated induction of mitophagy results in the depletion of CLDN5.** **A** Western blot analysis of ZO-1, occludin, CLDN5, and LC3 protein levels in hBMECs over 0–6 h of rEno stimulation. β-Tubulin was used as the loading control. **B** EB permeation assay in hBMEC monolayers treated with rEno-WT or vehicle, with or without the overexpression of CLDN5. Data are mean ± s.d.; *n *= 3. Significant differences were identified using a two-way ANOVA followed by Dunnett’s multiple comparisons test. **, *P* < 0.01. **C** Representative confocal microscopy images of colocalization between CLDN5 and HSP60 in hBMECs. Nuclei were stained with DAPI. Scale bar: 5 μm. **D** Representative confocal images showing localization between CLDN5 and LAMP1 in hBMECs pretreated with Mdivi-1 or BafA1 followed by the addition of rEno-WT. Nuclei were stained with DAPI. Scale bar: 5 μm. **E** Pearson’s correlation coefficient analysis of CLDN5 and LAMP1 colocalization from panel D. Data are mean ± s.d.; *n *= 50 cells from three independent experiments. Significant differences were identified using Kruskal–Wallis test. ***, *P* < 0.001. **F** Western blot analysis of HSP60, TOM20, CLDN5, and LC3 in hBMECs pretreated with or without Mdivi-1 before vehicle or rEno-WT treatment. β-Tubulin was used as the loading control. **G** Western blot analysis of CLDN5 in hBMECs treated with vehicle, rEno-WT or rEno-ΔH2. β-Tubulin was used as the loading control. **H** Western blot analysis of CLDN5 in wild-type (WT) and PINK1 knockout (PINK1 KO) hBMECs, with or without rEno-WT treatment. β-Tubulin served as the loading control. **I** Western blot analysis of HSP60, TOM20, CLDN5, and LC3 in hBMECs infected with wild-type (WT), *eno*::*kan*, or *eno*-ΔH2 strains. Uninfected cells served as control. β-Tubulin was used as loading control. **J** Immunohistochemical analysis of the protein level of CLDN5 in brain tissues from mice infected with the WT strain or the eno-ΔH2 mutant strain, as well as from uninfected control mice. Scale bar: 50 μm.

In view of the central role of tight junction proteins including ZO-1, occludin, and CLDN5 in maintaining BBB structural integrity [30], we examined the effect of rEno-WT on tight junction protein levels. rEno-WT markedly reduced the protein abundance of CLDN5, whereas no significant changes in the total protein levels of ZO-1 or occludin were detected (Figure 7A). Significantly, the overexpression of CLDN5 can effectively mitigate the disruption of BBB induced by rEno-WT (Figure 7B), which provides key clues for elucidating the molecular mechanism of Eno-mediated BBB damage. CLDN5 degradation can occur via ubiquitin–proteasome pathways [31]. However, the rEno-induced depletion of CLDN5 was blocked by the autophagy inhibitors 3-MA and BafA1 but not by the proteasome inhibitor MG132, indicating that CLDN5 is degraded via autophagy triggered by rEno rather than through upstream transcriptional downregulation (Additional file 7). Proteins gathering in mitochondria can be degraded by mitophagy [32], and CLDN5 is colocalized with mitochondria in cardiomyocytes [33]. We also found that a part of CLDN5 showed colocalization with HSP60 in hBMECs (Figure 7C), so we thought CLDN5 aggregating in mitochondria was degraded with Eno-induced mitophagy. Immunofluorescence imaging showed an enhanced localization of CLDN5 within lysosomes following treatment with rEno-WT, a phenomenon that was inhibited by the administration of Mdivi-1 and BafA1 (Figure 7D and E). Importantly, pretreatment of hBMECs with Mdivi-1 effectively inhibited the rEno-induced degradation of CLDN5 (Figure 7F). Likewise, the deletion of H2 in Eno, responsible for inducing mitophagy, resulted in a complete loss of its ability to deplete CLDN5 (Figure 7G). Upon knocking out PINK1, the reduction in CLDN5 levels induced by rEno was entirely mitigated (Figure 7H). Consistently, infection with the *eno*-ΔH2 strain restored HSP60 and TOM20 levels, reduced the LC3-II/LC3-I ratio, and attenuated CLDN5 depletion compared with WT strain infection (Figure 7I). Immunohistochemical analysis of brain tissues revealed a reduced protein level of CLDN5 in mice infected with the WT strain, as compared to uninfected mice. However, the reduction in CLDN5 level was attenuated in mice infected with the *eno*-ΔH2 strain (Figure 7J). These evidences proved that CLDN5 can be degraded via Eno-induced mitophagy.

## Discussion

**Figure 8:**
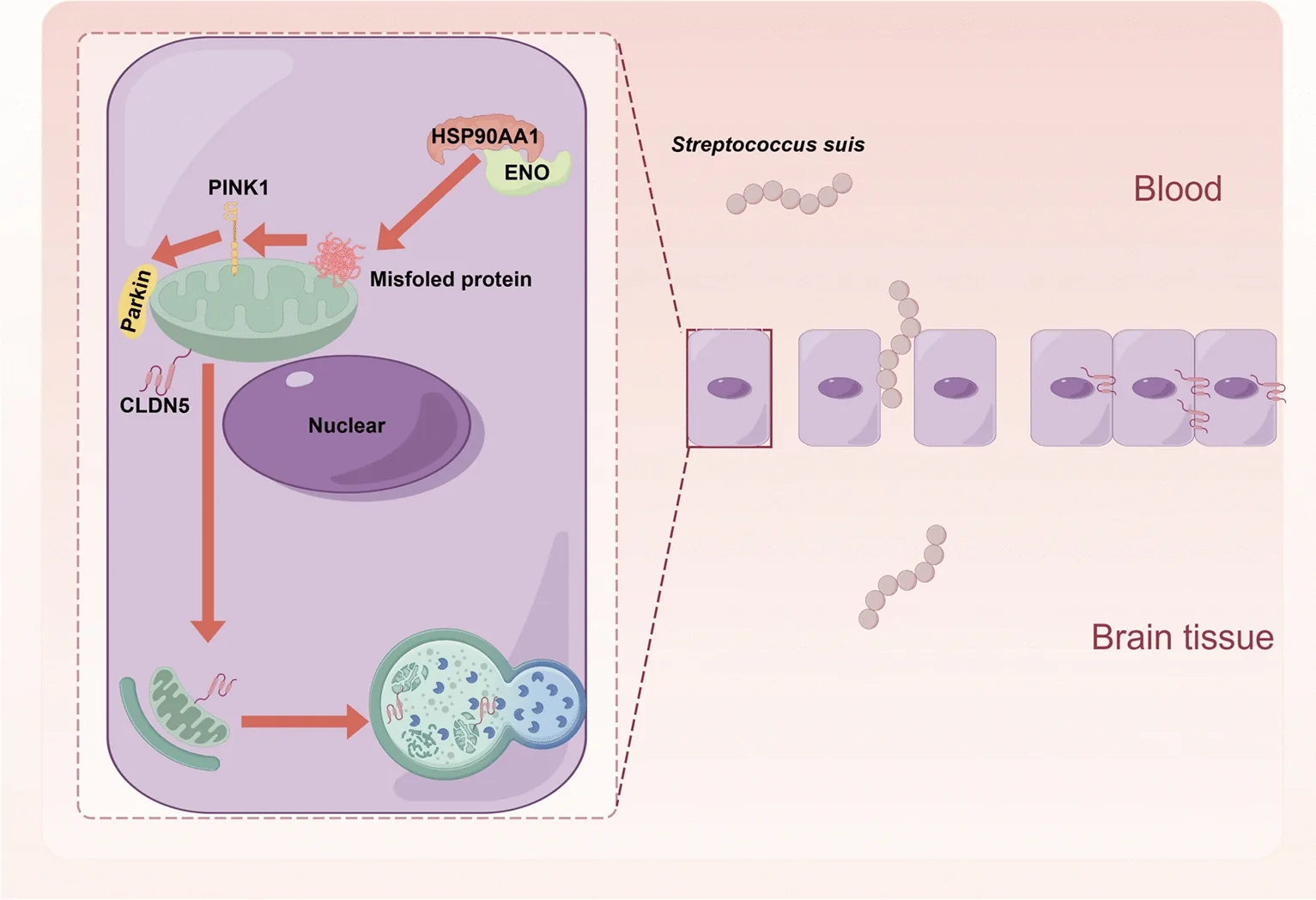
**Diagram summarizing the mechanism by which Eno induces mitophagy, leading to the disruption of the BBB. SS2 enolase interacts with host HSP90AA1 via its N-terminal second α-helix (residues 87–98). **This interaction potentially impairs HSP90AA1 function, causing misfolded protein accumulation in mitochondria. The proteostasis stress activates the PINK1-Parkin pathway, inducing mitophagy. Eno-triggered mitophagy leads to the degradation of the tight junction protein CLDN5 in brain endothelial cells, disrupting BBB integrity and facilitating bacterial meningitis.

The interaction between bacteria and mitophagy is a dynamic and complex process that involves various strategies employed by pathogens to manipulate or evade host cellular defenses. Understanding these interactions provides valuable insights into bacterial pathogenesis and the potential for developing therapeutic interventions targeting mitophagy pathways. In this study, we identify a connection between SS2 infection and mitophagy, with the activation of this autophagic response being contingent upon the Eno-HSP90AA1 interaction. Our findings indicate that mitophagy induced by sublethal concentration of Eno facilitates the degradation of CLDN5, thereby compromising cell tight junctions and increasing the permeability of the BBB (Figure 8).

Previous studies have indicated that high concentrations of Eno can compromise the integrity of the BBB by inducing apoptosis [12]. But our research demonstrates that even at sublethal concentration, Eno can still damage the BBB through the induction of mitophagy. This suggests a potential interplay between mitophagy and apoptosis, as mitophagy can inhibit apoptosis [34]. Furthermore, experiments involving mouse model of SS2 infection revealed that the inhibition of mitophagy significantly prevented Eno-induced BBB disruption. Although the physiological Eno concentration at the BBB during in vivo SS2 infection is unknown, these findings suggest that the local concentration of Eno within the infection microenvironment may not be sufficient to trigger host cell apoptosis. Instead, Eno can exerts its contributory effects on the disruption of BBB through the induction of mitophagy.

The PINK1-Parkin pathway is a crucial mechanism for maintaining mitochondrial quality through mitophagy, which is particularly significant in various physiological and pathological processes [35, 36]. Some studies have demonstrated that the PINK1-Parkin pathway can be activated in response to bacterial infections. The vacuolating toxin A (VacA), a critical virulence factor of *Helicobacter pylori*, interacts with various mitochondrial proteins, resulting in the dissipation of MMP, which subsequently activates the PINK1-Parkin-dependent mitophagy [37]. Similarly, *Mycobacterium bovis* induces mitophagy via the PINK1-Parkin pathway, but the specific virulence factors responsible for this process remain unidentified [38]. These processes are dependent on the MMP dissipation, but it is not the inducer for Eno-induced mitophagy. The misfolded protein in mitochondria can activate PINK1, which then recruits Parkin to initiate mitophagy [27]. Our research illustrates that this process can be exacerbated by bacterial toxins. Eno interacts with HSP90AA1, a process that potentially drives the accumulation of misfolded proteins in mitochondria and subsequently impairs mitochondrial import function to initiate mitophagy. But in this study, we only demonstrate that Eno-induced mitophagy is not primarily initiated by the change of MMP. There is still a possibility that Eno-induced mitophagy requires both the interaction of Eno with HSP90AA1 and MMP dissipation. Eno is located within the bacterial cytoplasm and on the cell surface, but the mechanism by which it enters host cells remains unclear. Utilizing ultracentrifugation and fluorescent tracking, Shi et al. verified that Eno is highly enriched in SS2 membrane vesicles (MVs) and is efficiently internalized by host endothelial cells [22]. Based on this experimental evidence, we propose that SS2 facilitates the delivery of Eno into the host cytoplasm through the release of MVs. Because enolase is highly conserved among streptococci and related non-pathogenic lactic acid bacteria, the interaction between internalized Eno and the ubiquitous host chaperone HSP90AA1 might represent a general host stress response to ectopic enolase, rather than a sequence-specific virulence trait unique to SS2.

As a crucial molecular chaperone, HSP90AA1 plays a significant role in cellular signal transduction and stress response mechanisms by modulating the conformational maturation, stability, and functional activation of client proteins [39]. The VP2 protein of the avian double-stranded RNA virus interacts with HSP90AA1 to deactivate the Akt/mTOR signaling pathway, thereby inducing autophagy [40]. HSP90AA1 is also involved in maintaining mitochondrial cristae structure through its interaction with OPA1 [41]. Here we reported a new function of HSP90AA1 connecting mitochondria regulation and autophagy. The formation of the Eno-HSP90AA1 may promote the mitochondrial accumulation of misfolded proteins, potentially via inhibiting HSP90AA1 function. While targeted inhibition of mitochondrial isoform of HSP90 (TRAP1) has been shown to induce misfolded proteins aggregation [42, 43], it is possible that HSP90AA1 exhibits analogous functions. Cytosolic protein aggregates can be sequestered by mitochondria, impairing their function [44, 45]. Consequently, it is also reasonable that misfolded proteins in mitochondria originate from the cytosol, where a significant portion of HSP90AA1 is localized [46]. HSP90AA1 is known to interact with various client proteins, enhancing their stability and prolonging their half-life [47–49]. Inhibition of HSP90AA1 function may precipitate the degradation of essential proteins responsible for proper protein folding, which facilitates mitophagy. Although further investigation is required to fully understand these processes, this discovery broadens the mechanism of mitophagy induced by pathogenic bacteria and offers novel insights for the treatment of SS2 infections.

Functional variability of mitophagy influences the interaction between bacterial pathogens and their hosts. During mitochondrial oxidative phosphorylation, electron leakage from the electron transport chain generates mitochondrial reactive oxygen species (mtROS), which are crucial for host defense against infections by oxidizing and damaging microbial membrane structures, nucleic acids, and functional proteins [50, 51]. Pathogens such as *Burkholderia pseudomallei*, *Listeria monocytogenes*, *Yersinia pestis*, and *Neisseria gonorrhoeae* can effectively diminish intracellular mtROS levels by inducing mitophagy, thereby impairing the host immune clearance capabilities and enhancing the intracellular survival advantage of the bacteria [17, 18, 52, 53]. *Mycobacterium bovis*-induced mitophagy competitively utilizes phosphorylated TBK1, which leads to reduced TBK1 availability for xenophagy, thereby inhibiting xenophagy-mediated bacterial clearance within the host [38]. However, mitophagy functions not only as a "defense shield" for pathogen survival but also as a "molecular engine" facilitating bacterial dissemination. *Brucella*-induced mitophagy does not directly influence the intracellular proliferation of bacteria, but it enhances bacterial cell-to-cell transmission and tissue invasion by modulating mitochondrial *Brucella*-containing vacuoles [19]. The investigation into Eno-induced mitophagy also supports this transformative perspective. Our research demonstrates that this process can substantially compromise the integrity of the BBB, independent of Eno-mediated apoptosis. CLDN5 is a key component to form tight junction, which is important for integrity of the BBB, and the decline of CLDN5 protein level is associated with comprised BBB [54, 55]. Serine/threonine kinase from SS2 affects the expression of the E3 ubiquitin ligase HECTD1, which in turn facilitates the degradation of CLDN5 [56]. This study elucidates a novel mechanism by which SS2 induces the degradation of CLDN5. This is the first evidence that pathogenic bacteria can contribute to the disruption of BBB integrity via mitophagy, unveiling a novel paradigm in which pathogens exploit mitochondrial quality control mechanisms to achieve neural invasion.

Finally, we acknowledge certain limitations in the present study that warrant further investigation. First, from a mechanistic perspective, the essential nature of HSP90AA1 for basic cell viability precluded the use of standard knockout or knockdown models, since significant depletion could induce severe baseline proteotoxic stress and spontaneous mitophagy, thereby confounding the specific effects of Eno on CLDN5 degradation. While our overexpression experiments strongly support this molecular axis, future studies employing conditional knockdown models or targeted interaction-blocking peptides are required for comprehensive genetic validation. Second, regarding our in vivo meningitis model, although the intraperitoneal injection route reliably induces the sustained bacteremia necessary for SS2 central nervous system invasion, its initial peritoneal absorption phase may not perfectly replicate the immediate hematogenous interactions at the BBB endothelial interface provided by an intravenous inoculation. The modest in vivo attenuation observed in our mutant models thus suggests that this Eno-mediated mitophagy pathway acts as a contributory mechanism, likely operating in concert with other established virulence factors to fully achieve BBB disruption. Lastly, because the exact physiological concentration of Eno during in vivo infection remains undetermined, our study was constrained to using 2 µg/mL rEno. While this treatment successfully induces relevant biological effects, it represents an approximation that may not fully capture the true, multifaceted infection microenvironment.

## Conclusions

In conclusion, Eno from SS2 engages with HSP90AA1, which may impair host chaperone function and activate the PINK1-Parkin pathway through the disruption of proteostasis, ultimately leading to the induction of mitophagy. This autophagic mechanism results in the degradation of CLDN5, offering innovative insights into the contributory role of Eno in mediating BBB disruption. This understanding provides a theoretical foundation for the development of innovative anti-infective strategies.

## Supplementary Information

**Additional file 1. The** ***eno::kan*** **and** ***eno*-ΔH2 mutant strains exhibit no significant differences in growth kinetics and cellular invasion capacities compared to the WT strain.** (A)  Western blot analysis of Eno in the WT, *eno::kan*, and *eno*-ΔH2 strains. (B) In vitro growth curves of the WT, *eno::kan*, and *eno*-ΔH2 strains. (C) The whole blood survival of WT, *eno::kan*, and *eno*-ΔH2 strains. (D) Intracellular bacterial loads of WT, eno::kan, and eno-ΔH2 strains in hBMECs.

**Additional file 2. Eno induces the formation of mito-lysosome in SS2 infection.** (A) Representative confocal microscopy images of hBMECs stably expressing the mito-QC reporter, following infection with the WT strain and the *eno::kan* mutant strain for 4 hours. Scale bar: 5 μm. (B) Quantification of the number of mito-lysosome per cell in panel A. *n* = 51 cells from three independent experiments. Significant differences were identified using Kruskal–Wallis test. ***, *P* < 0.001.

**Additional file 3. Eno enters cytoplasm and partially colocalizes with mitochondria.** Confocal microscopy images of hBMECs with or without SS2 infection. Cells were probed with anti-Eno and anti-TOM20 antibodies. Nuclei were stained with DAPI. Scale bar: 5 μm.

**Additional file 4. Eno directly interacts with HSP90AA1.** Pull-down validation of the direct interaction between rEno-WT/rEno-ΔH2 and rHSP90AA1.

**Additional file 5. Mitophagy does not affect intracellular survival of SS2 in hBMECs.** Intracellular survival of SS2 in hBMECs pretreated with or without Mdivi-1. Data are mean ± s.d.; *n* = 3.

**Additional file 6. 2 µg/mL rEno does not show cytotoxicity or trigger apoptosis.** (A) LDH levels released by hBMECs following treatment with 2 µg/mL rENO-WT or rEno-ΔH2 for 4 hours or 6 hours. Data are mean ± s.d.; *n* = 3. (B) Flow cytometry analysis of hBMECs treated with 2 µg/mL rENO-WT or rEno-ΔH2 for 6 hours to detect apoptosis via FITC-Annexin V/propidium iodide staining.

**Additional file 7. CLDN5 is degraded via autophagy.** Western blot analysis of CLDN5 in hBMECs pretreated with 3-MA, BafA1 or MG132 before vehicle or rEno-WT treatment. β-Tubulin was used as the loading control.

**Additional file 8. Details of primers applied in this study.**

## Data Availability

The mass spectrometry proteomics data have been deposited in the iProX repository (https://www.iprox.cn/) with the dataset identifier IPX0012733000.

## Abbreviations

SS2: *Streptococcus suis* Serotype 2
BBB: the blood–brain barrier
Eno: Enolase
MMP: mitochondrial membrane potential
hBMECs: human brain microvascular endothelial cells
PINK1: PTEN induced kinase 1
Parkin: Parkin RBR E3 ubiquitin protein ligase
HSP90AA1: heat shock protein 90 alpha family class A member 1
CLDN5: claudin-5
TOM20: translocase of outer membrane 20
HSP60: heat shock protein HSP60
LAMP1: lysosomal associated membrane protein 1
LONP1: lon peptidase 1
