# Supplementary material for: Streptococcus suis-induced mitophagy via Eno-HSP90AA1 interaction disrupts the blood–brain barrier
Source: Vet Res. 2026 Nov 7;57:197. doi: 10.1186/s13567-026-01860-5 (PMC13744109; doi:10.1186/s13567-026-01860-5)
Supplement: Supplementary file 1 [file 13567_2026_1860_MOESM8_ESM.docx]

**Table S1** Details of primers applied in this study.

| Primer name | Sequence |
| --- | --- |
| 3HA-F | cgcaagcttatgtcaattattactgatgtttacgctcgc |
| 3HA-R | cgcggatccttattttttcaagttgtagaatgagttcaagcctttgta |
| 28a-F | cgcgctagcatgtcaattattactgatgtttacgctcgc |
| 28a-R | cgcggatccttattttttcaagttgtagaatgagttcaagcctttgta |
| 3HA-ΔN-terminus-F | cgcaagcttactccaatgatgaacatcatcaacgg |
| 3HA-ΔN-terminus-R | cgcggatccttattttttcaagttgtagaatgagttcaagcctttgt |
| 3HA-ΔC-terminus-F | cgcaagcttatgtcaattattactgatgtttacgctcgc |
| 3HA-ΔC-terminus-R | cgcggatcctggcaatactttagtgttgaatccacc |
| 3HA-ΔS-F | cgcaagcttgttccttcaggagcttctactggt |
| 3HA-ΔS-R | cgcggatccttattttttcaagttgtagaatgagttcaagcctttgta |
| 28a-ΔS-F | cgcgctagcgttccttcaggagcttctactggt |
| 28a-ΔS-R | cgcggatccttattttttcaagttgtagaatgagttcaagcctttgta |
| ΔH1-F | ggtcttggtactcaaatcggttttgacgtt |
| ΔH1-R | aacgtcaaaaccgatttgagtaccaagacc |
| ΔH2-F | tttgacgttcgtgatggtactcctaacaaa |
| ΔH2-R | tttgttaggagtaccatcacgaacgtcaaa |
| ΔH3-F | aaaggtaaattgggtcttgaagtgccactt |
| ΔH3-R | aagtggcacttcaagacccaatttaccttt |
| ΔH4-F | taccttgaagtgccaggattcaacactaaa |
| ΔH4-R | tttagtgttgaatcctggcacttcaaggta |
| Δsly-A | cgcggatccccttcctcaaaggttgaaatatctgcca |
| Δsly-B | acatgaaaggataaaactttctggcaatgtattatact |
| Δsly-C | agtataatacattgccagaaagttttatcctttcatgt |
| Δsly-D | cgcaagcttcaggcgaagatatttgttatctaggggat |
| eno1-A | cgcggatccgtgcgtagggagattgtcctattttttcat |
| eno1-B | catcttgttcaatcatgtcgatagcttgttga |
| KAN-C | tcaacaagctatcgacatgattgaacaagatg |
| KAN-D | gatcatagcgcggaagaactcgt |
| eno2-E | gacgagttcttccgcgctatgatc |
| eno2-F | cgcaagcttttattttttcaagttgtagaatgagttcaagcctttgta |
| Δeno-H2-A | cgcggatcc gtgcgtagggagattgtcctattttttcat |
| Δeno-H2-B | ctttgttaggagtaccatcacgaacgtcaaaa |
| Δeno-H2-C | ttttgacgttcgtgatggtactcctaacaaag |
| Δeno-H2-D | cgcaagcttttattttttcaagttgtagaatgagttcaagcctttgta |
